# Dissociating Relational and Conjunctive Binding in Working Memory: A Case Study of Hippocampal Damage

**DOI:** 10.1002/alz70856_096430

**Published:** 2025-12-24

**Authors:** Freddie O'Donald

**Affiliations:** ^1^ NHS Tayside, Dundee, United Kingdom; University of Edinburgh, Edinburgh, United Kingdom

## Abstract

**Background:**

Working memory (WM) allows for the temporary storage and processing of information, and the construct of binding enables the integration of such information into a coherent whole. Binding can be categorised into relational binding (associating separate features, such as shape and colour) and conjunctive binding (integrating features within a single object). Relational binding is thought to depend on hippocampal function, whereas conjunctive binding relies on parahippocampal structures. This case study examines WM binding functions in a young adult with hippocampal damage to explore the implications for understanding WM processes and early Alzheimer's disease (AD) detection.

**Method:**

Patient AS, a 21‐year‐old university student with bilateral hippocampal hypoxia, underwent a detailed neuropsychological assessment. WM binding tasks included multiple visual arrays designed to target both (1) relational binding, requiring the pairing of shapes and colours presented separately, and (2) conjunctive binding, requiring the recognition of coloured shapes as unified objects. Additional tasks assessed span capacity for individual features (Symbol Span from the WMS‐IV) and visuospatial memory (Visual Reproduction I and II from the WMS‐IV). Structural imaging evaluated the integrity of hippocampal and parahippocampal regions, with a specific focus on the perirhinal cortex and hippocampal atrophy.

**Result:**

AS demonstrated severe impairment on tasks reliant on relational binding, whereas performance on conjunctive binding tasks fell within the typical range. Performance on span tasks and single‐feature recognition suggested the absence of generalised WM deficits. Imaging confirmed bilateral hippocampal atrophy, with preservation of parahippocampal regions, including the perirhinal cortex. These findings align with the hypothesis that relational binding is hippocampus‐dependent, whereas conjunctive binding relies on alternative neural networks, such as the ventral visual stream.

**Conclusion:**

This case demonstrates a dissociation between relational and conjunctive binding in WM, supporting the role of the hippocampus in relational feature associations and the reliance of conjunctive binding on parahippocampal structures. These findings demonstrate the utility of conjunctive binding tasks for the early detection of Alzheimer's disease‐related dementia. Unlike traditional relational binding tasks, which may remain intact until later stages of hippocampal involvement, conjunctive binding tasks could act as sensitive markers of subhippocampal dysfunction in the earliest stages of AD‐related pathology.

